# An Ectopic ACTH Secreting Metastatic Parotid Tumour

**DOI:** 10.1155/2016/4852907

**Published:** 2016-01-20

**Authors:** Thomas Dacruz, Atul Kalhan, Majid Rashid, Kofi Obuobie

**Affiliations:** ^1^University Hospital of Wales, Cardiff, UK; ^2^Department of Diabetes & Endocrinology, Royal Glamorgan Hospital, Mid Glamorgan CF72 8XR, UK; ^3^Royal Gwent Hospital, Newport, UK

## Abstract

A 60-year old woman presented with features of Cushing's syndrome (CS) secondary to an ectopic adrenocorticotropic hormone (ACTH) secreting metastatic parotid tumour 3 years after excision of the original tumour. She subsequently developed fatal intestinal perforation and unfortunately died despite best possible medical measures. Ectopic ACTH secretion accounts for 5–10% of all patients presenting with ACTH dependent hypercortisolism; small cell carcinoma of lung (SCLC) and neuroendocrine tumours (NET) account for the majority of such cases. Although there are 4 previous case reports of ectopic ACTH secreting salivary tumours in literature, to our knowledge this is the first published case report in which the CS developed after 3 years of what was deemed as a successful surgical excision of primary salivary tumour. Our patient initially had nonspecific symptoms which may have contributed to a delay in diagnosis. Perforation of sigmoid colon is a recognised though underdiagnosed complication associated with steroid therapy and hypercortisolism. This case demonstrates the challenges faced in diagnosis as well as management of patients with CS apart from the practical difficulties faced while trying to identify source of ectopic ACTH.

## 1. Background

Cushing's syndrome (CS) is associated with a constellation of signs and symptoms related to hypercortisolism. Common conditions such as obesity, chronic alcoholism, and depression share clinical and phenotypic features which overlap with those seen in patients with CS; often it results in delayed investigations and management for the patients.

True CS can either be ACTH dependent or ACTH independent [1]. ACTH dependent CS is uncommon with 1-2 cases/million of population/per year reported in the literature, with pituitary adenoma being source of ACTH in two-thirds of such patients [1]. SCLC and pancreatic/thymic/bronchial NETs remain the commonest tumours associated with ectopic ACTH secretion [2–4]. There are rare reports linking medullary carcinoma of thyroid, phaeochromocytoma, and ovarian and salivary tumours with ectopic ACTH secretion [5, 6]. To our knowledge, there are only 4 previous case reports of ectopic ACTH secretion from a salivary gland carcinoma leading to CS [7–10]. This is also the first report of an ectopic ACTH-secreting metastatic salivary tumour in which the CS developed after 3 years of complete and what was deemed as a successful surgical excision of primary salivary tumour.

## 2. Case Presentation

Mrs. XX, a 60-year-old woman was hospitalised with 5-month history of worsening fatigue, leg swelling, and difficulty in walking. Her past medical history included primary hypothyroidism and hypertension. She had been diagnosed with an acinic cell carcinoma of the left parotid gland, which was resected 3 years prior to current admission. She was under follow-up of the surgical team with no obvious evidence of disease recurrence or relapse at the time of presentation.

On examination in medical assessment unit, she had florid features of CS including central obesity, plethoric face, skin thinning, purplish abdominal striae, and proximal muscle weakness. Her blood pressure was elevated with rest of the general physical and systemic examination being unremarkable.

## 3. Investigations

Her initial investigations results were as shown in Table 1.

On the basis of clinical suspicion of hypercortisolism, she underwent further tests as shown in Tables 2 and 3.

*Magnetic Resonance Image of the Pituitary*. It showed normal pituitary gland.

*Abdomen/Pelvis/Thorax CT*. It shows a lytic lesion on the left ischium bone suggestive of a metastatic carcinoma. A collection of gas around the sigmoid colon was noticed which was suggestive of perforated sigmoid diverticulitis.

*Histopathology*. Biopsy of ischial lesion showed features consistent with a metastatic poorly differentiated acinic cell carcinoma with negative staining for ACTH (Figure 2). However, the previously resected primary parotid tumour of acinic cell carcinoma were stained positively for ACTH.

## 4. Treatment

Our patient was commenced on metyrapone therapy on day 6 of admission with a gradual uptitration of the dose. The course of her disease was aggressive with subsequent development of intestinal perforation. Interestingly, she had minimal symptoms and signs on clinical examination suggestive of intestinal perforation with this diagnosis being only established based on the radiological investigations.

She later developed sepsis and was managed in intensive care unit. Unfortunately, despite the best possible care her condition continued to deteriorate and she died due to complications related to her ectopic ACTH related CS secondary to a metastatic salivary gland tumour.

## 5. Discussion

Ectopic ACTH secreting tumours are rare with a reported prevalence of 8–18% of all the patients with CS [11, 12]. The SCLC along with NETs (bronchial, thymic, and pancreatic) remains the commonest ACTH secreting tumours [2–4]. Our patient presented with CS secondary to an ectopic ACTH producing metastatic parotid tumour. In our literature search, we could only identify 4 previously reported cases of metastatic salivary tumour associated with ectopic ACTH production [5–8]. In most of the previous case reports, CS was diagnosed either at the time of diagnosis of primary salivary tumour or within months of commencement of treatment (median duration of CS diagnosis 6 months after initial salivary tumour was detected). Our patient is the first case report of an ectopic ACTH secreting salivary tumour presenting with a distant metastasis after over 3 years of being deemed cured of primary tumour (postsurgical excision).

Our patient presented with 5-month history of nonspecific features including weight gain, malaise, and leg swelling. There was a possible delay in her investigations and management due to nonspecific nature of her symptoms. An overlap of such symptoms is not uncommonly seen in obesity, depression, and chronic alcoholism which have a much higher prevalence in clinical practice.

On admission, our patient had florid signs of CS including proximal myopathy and purple striae which are considered relatively specific for CS [13]. The initial screening (24 hour UFC and ODST) tests were suggestive of hypercortisolism. An unsuppressed cortisol level on LDDST further established the biochemical evidence for CS. Elevated ACTH levels pointed towards possibility of a pituitary or ectopic source of ACTH/CRH. The MRI of pituitary gland was reported as normal in our patient. In clinical practice, it is often challenging to distinguish an ectopic ACTH secreting tumour from a pituitary source especially considering that in 40% of CD patients a MRI pituitary may be normal [14]. Inferior petrosal sinus sampling (IPSS) was considered as the next step in investigation although it could not be carried out as our patient was deemed medically unfit to undergo this invasive procedure. Previously, a HDDST was considered to be a useful additional test to distinguish pituitary from an ectopic source of ACTH. In CD, a cortisol suppression of > 50% from the basal level is noticed in only 80% of the patients undergoing HDDST, limiting the diagnostic usefulness of this test [15]. IPSS remains the gold standard to distinguish pituitary from an ectopic source of ACTH [16].

The ectopic ACTH source may not be obvious initially in patients with well-differentiated neuroendocrine tumours as these are generally slow growing and may not be visualised on routine radiological imaging. In 12% of patients, despite extensive investigations, the source of ectopic ACTH may not be identified [17]. Various modalities have been suggested to investigate and identify source of ectopic ACTH secretion including use of Somatostatin scintigraphy [18]. Proopiomelanocortin (POMC) serves as precursor molecule to ACTH and undergoes posttranslational proteolytic processing in corticotrophs. This proteolysis processing is mediated by serine proteinases such as PC1, PC2, and PC3 which are expressed only in the pituitary gland and play a role in POMC cleavage. Ectopic ACTH tumours are characterised by an abnormal circulating ACTH precursor to ACTH ratio as compared to ACTH secreting pituitary adenoma due to a possible aberrant POMC processing [19, 20]. As no single imaging technique is believed to have optimal accuracy for localisation of ectopic ACTH secreting tumour, it is recommended to combine more than one imaging modality such as conventional CT along with somatostatin scintigraphy scan [18].

A whole body CT scan in our patient revealed a lytic lesion on the left ischial bone and patient went on to develop bowel perforation, with minimal features. Exogenous steroid use and CS have been associated with increased risk of intestinal perforation and sepsis [21, 22]. It is not uncommon for the cortisol excess to mask signs associated with intestinal perforation delaying the diagnosis which can potentially prove fatal as in our patient.

**Figure 1:**
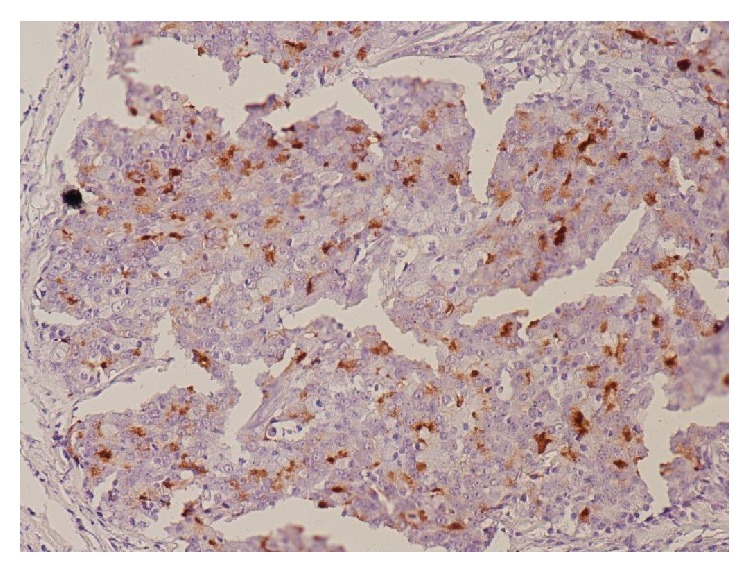
Immunohistochemical stain (×400) for ACTH showing tumour cells being positive (brown).

**Figure 2:**
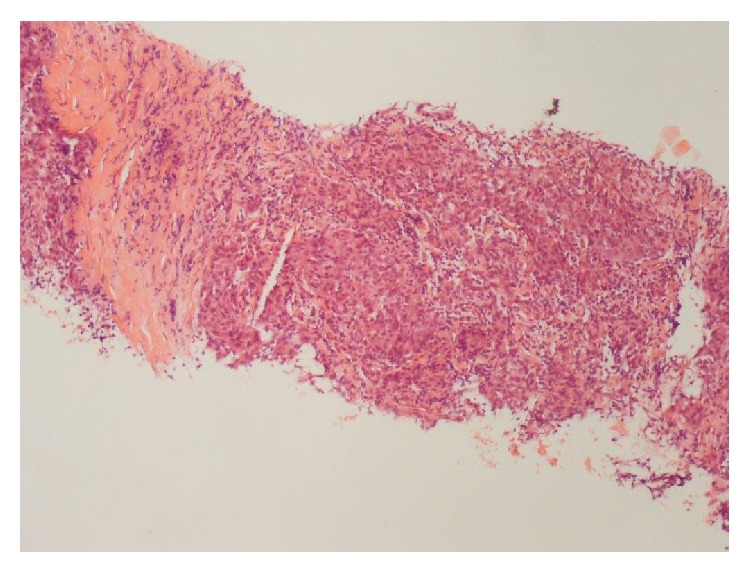
Haematoxylin and Eosin stain (×200) bony metastasis with poorly differentiated malignant epithelial cells seen.

The ischial lytic bone lesion on histopathological analysis was confirmed to be a metastasis from the parotid gland tumour although it was stained negatively for ACTH. The immunostaining of preserved original tumour tissue was stained positively with ACTH confirming the diagnosis (Figure 1). Negative ACTH staining in ectopic tumour tissue is believed to be associated with a more aggressive disease course and a worse prognosis [23]. It is postulated that the tumour which has a high secretory rate gets depleted of all intracellular ACTH which results in negative immunostaining on histopathological analysis as may have been the case in our patient.

The further investigations and treatment were limited in our patient because of her rapid clinical deterioration. In retrospective analysis, we acknowledge the fact that there was a relative delay in initiation of metyrapone therapy in her case. Bilateral adrenalectomy should be considered as a life-preserving treatment option in patients presenting with severe ACTH dependent CS uncontrolled with medical therapy or in patients with metastatic ectopic ACTH secreting tumours [24] although our patient was deemed an unsuitable candidate for any surgical intervention.

In summary, our patient presented with a highly aggressive ectopic ACTH secreting metastatic parotid tumour. Earlier suspicion and management of hypercortisolism could have potentially improved her prognosis although it may or may not have altered the eventual outcome.

## Supplementary Material

Supplementary figure showing cortisol level fluctuations despite metyrapone therapy.

## Figures and Tables

**Table 1:** Initial routine blood test results.

Investigations	Results	Reference range
Haemoglobin	125 g/L	115–155
White blood cell count	11.5 × 10^9^/L	4–10.5
Neutrophils	7.5 × 10^9^/L	1.5–7.5
Platelets	318 × 10^9^/L	150–450
Serum sodium	141 mmol/L	135–145
Serum potassium	2.6 mmol/L	3.5–5.5
Serum urea	3.6 mmol/L	2.5–7.8
Serum creatinine	51 *μ*mol/L	50–100
Fasting blood glucose	7 mmol/L	

**Table 2:** Further investigations.

Investigations	Results	Reference range
Prolactin	429 mU/L	<560
Insulin-like growth factor 1	11 nmol/L	12.0–54.0
24-hour urinary cortisol level	4481 nmol	<146
ACTH	106 ng/L	7–63

**Table 3:** Dynamic tests to assess hypercortisolism.

Investigation	Procedure	Timing of the test	Results
Overnight dexamethasone test (ODST)	1 mg dexamethasone given at midnight	Measurement of 9 am cortisol	785 nmol/L
Low dose dexamethasone test (LDDST)	0.5 mg dexamethasone given every six hours for 48 hours	Cortisol measured after last dose of dexamethasone	577 nmol/L

## Learning Points

- The signs and symptoms associated with Cushing's syndrome may be nonspecific which can potentially lead to a delay in diagnosis and management.
- The presence of proximal myopathy, easy bruisability, and purple striae should prompt investigations for CS as untreated disease carries high morbidity and mortality.
- Distinguishing pituitary from ectopic source of ACTH is challenging especially if there is no obvious adenoma identified on magnetic resonance imaging (MRI) of the pituitary. This is a recognised clinical scenario in up to 40% of the patients with Cushing's disease (CD).
- SCLC and NETs remain the commonest tumours associated with ectopic ACTH secretion although it has been reported in association with salivary gland, medullary carcinoma of thyroid, phaeochromocytoma, and ovarian tumours.
- Bilateral adrenalectomy should be considered as a life-preserving treatment option in patients presenting with severe ACTH dependent CS uncontrolled with medical therapy or in patients with metastatic ectopic ACTH secreting tumours.
