# Supplementary figures and images for: An Ectopic ACTH Secreting Metastatic Parotid Tumour
Source: Case Rep Endocrinol. 2016 Jan 20;2016:4852907. doi: 10.1155/2016/4852907 (PMC4745306; doi:10.1155/2016/4852907)

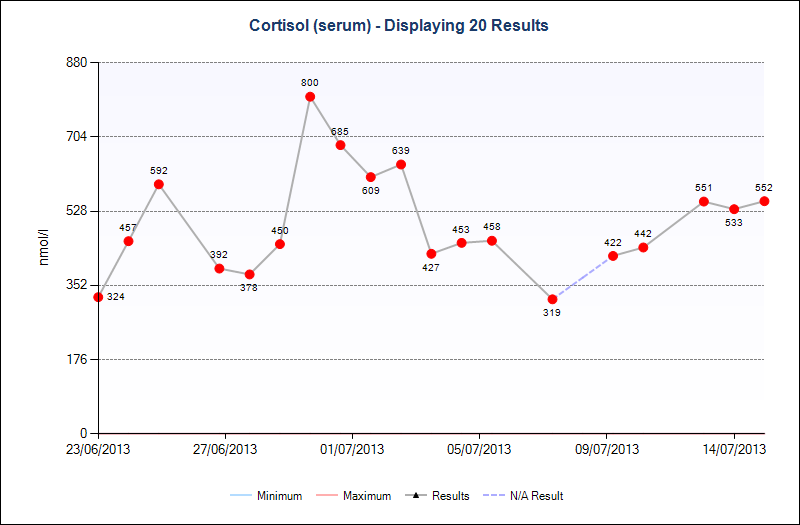

Supplement: Supplementary file 1 — Supplementary figure showing cortisol level fluctuations despite metyrapone therapy. [file 4852907.f1.docx]
